# Household costs of seeking outpatient care in Egyptian children with diarrhea: a cross-sectional study

**DOI:** 10.11604/pamj.2013.14.42.1989

**Published:** 2013-01-29

**Authors:** Abeer Barakat, Eman Fawzy Halawa

**Affiliations:** 1 Department of public health and epidemiology, Faculty of Medicine, Cairo University, Egypt; 2 Department of pediatrics, Faculty of Medicine, Cairo University, Egypt

**Keywords:** Economic burden, diarrhea, Egypt

## Abstract

**Introduction:**

Addressing difficulties of seeking and getting health care would lower the burden of diarrhea among ill children from developing countries as Egypt. The purpose of the study is to evaluate the economic burden of diarrhea associated with outpatient visits of children in Egypt by identifying the different types of related costs.

**Methods:**

This cross-sectional clinic-based survey was done by interviewing parents of 763 children presenting with diarrhea to the outpatient clinics of Pediatric Hospital of Cairo University. Estimated costs included tangible costs (direct, indirect) and intangible costs (forms of suffering). Insurance status of the children was also described. Descriptive statistics were presented in frequency tables, median, minimum, maximum, interquartile range, mean and standard deviation, whenever appropriate.

**Results:**

It was found that 90. 7% of the studied children were of low and middle socioeconomic standard with a median monthly family income of US$83 and a median monthly expenditure of LE US$79. The average direct and indirect costs of acute diarrhea per case were US$13.2±19.5 and US$11.3±93.1 respectively. The mean cost per diarrheal episode is US$24.5 which almost consumes 29.5% of the mean monthly income. About 61% of cases sought medical care before visiting our hospital, 43.6% of them visited more than one provider. Awareness about health insurance was found in 72.7% and coverage by a health insurance system in 33%. Of insured patients only 41.4% utilized the insurance services.

**Conclusion:**

Diarrhea causes great socio-economic burden for families in Egypt, which could result in significant delay in seeking health care.

## Introduction

Infections that cause diarrhea are a major public health problem in developing countries and other places where resources are scarce, particularly in young children. Approximately 15% of children die of diarrhea annually [1]. Gastrointestinal infections in children can significantly impact their families and society and result in increased medical expenditures, lost productivity, childcare and pain and suffering [2].

Resource-poor countries have long struggled to control infectious disease, reduce mortality and severe morbidity, and improve childhood survival rates with inadequate resources that are echoed in delayed diagnosis and poor service delivery at local levels. The direct costs for health care and medical services, and added indirect costs, deterred poor women from presenting with sick children. Those who eventually sought care often had to finance health spending through out-of-pocket payments and loans, or sold property, goods or labor to meet the costs. Costs were often catastrophic, exacerbating the extreme poverty of those least able to afford it [3].

Knowledge about the direct and indirect costs of acute diarrhea may provide the needed impetus for control of acute diarrhea in Egypt. It is therefore important to document these costs for formulating effective preventive health care policy.

The aim of this study is to evaluate the economic burden of diarrhea associated with outpatient visits of children in Egypt, including the direct costs of health care services for diarrhea, indirect costs and intangible costs to the individual patients. A complete picture on health care provision data and the pattern of their disease was also provided. The results of that survey would be useful in designing health plans and policies related to child empowerment.

## Methods

### Study setting

This cross-sectional quantitative clinic-based study was done at the outpatient clinics of Pediatric Specialized Hospital of Cairo University. This facility is the largest pediatric teaching referral hospital in Egypt with 600 beds. It provides both primary and tertiary care. The study was conducted during the peak of diarrheal season (from May to November, 2011).

### Study population

The caregivers accompanying children under 12 years presenting with acute diarrhea were interviewed by Cairo University Hospital (CUH) house officers. Diarrhea was defined as three or more loose bowel movements during a 24-h period [4]. A sample size of 1500 was intended to be included, however due to logistic reasons 799 were interviewed.

### Study tools

A structured questionnaire was developed and pilot-tested. It purposed to identify different types of costs related to seeking care for diarrhea among children. Estimated costs were tangible (direct, indirect) and intangible (including decreased daily activities, transport time and waiting time). Description of the insurance status of the children was also done. The items of the questionnaire included:

- Demographic characteristics of study population
- Pattern of their disease
- Health care seeking pattern
- Tangible direct costs of seeking of care for diarrheal disease including providers fees, medications, investigations and hospital fees
- Tangible indirect costs including transportation and missed work days. Intangible costs including transport time and waiting time
- Health insurance awareness, coverage, willingness to pay and for which package of services

All costs were calculated in 2005 US dollars and were reported as US dollars with use of an exchange rate of 6.03 LE to each US dollar.

### Research team

The House officers Research Core Team was initially trained on general concepts of research methodology, shared in developing, pilot-testing and finalizing the questionnaire. They were divided into 5 groups for data collection and entry, one leader for each. One field supervisor monitored their performance. Informed consent was obtained from the parents or the legal guardian of the children. In order to maintain patient confidentiality, no name or initials were used. The study was approved by the local institutional review board of pediatric department of Cairo University.

### Data analysis

The SPSS version 16 was used. Descriptive statistics were presented in frequency tables, median, minimum, maximum, interquartile range, mean and standard deviation, whenever appropriate.

## Results

During the 7 months study period which is the peak of diarrheal season (from May to November 2011), 799 parents of children with diarrhea were invited to participate in the study. Thirty six questionnaires were excluded due to lacked important data. Therefore, 763 questionnaires remained for analysis. The demographic characteristics of the study population are shown in Table 1. Of the studied population, those less than one year and 2years represented 39.6% and 79.5% respectively. About 60% were of middle socioeconomic standard, with a median monthly family income of US$83 and a median monthly expenditure of US$79.

**Table 1:** Demographic characteristics of study population

Determinants		%
Age(y)	≤5	95.3
	> 5	4.7
	Mean ±SD	2.1 ± 1.6
Sex	Males	55.9
Child order*	First	9.3
	Last	83.3 *
	≥ 4^th^	19.7 *
Socioeconomic level	Low	32.7
	Middle	58.1
	High	9.2
Family income/month (US$)	Mean ±SD	84 ± 83.6
	Median, interquartile range	83, 58 (116-58)
Family expenditure/month	Mean ±SD	99.5 ± 86
	Median, interquartile range	79, 66 (116-50)
Household possessions	≤ 10*	60.4
	>10	39.6

* household possessions reported by the World Bank in assessing poverty

The mean ±SD of diarrheal motions was 7.5 ± 5.4 per day. Diarrhea was recurrent in 54.9% and dehydration was found in almost 52.7% of cases. The mean ±SD of number of diarrheal episodes during the last year was 3.1 ± 3.9. Associated symptoms included vomiting, fever and decreased daily activities in 72.6%, 72.7% and 86.5% of cases respectively.

The Health care seeking pattern is shown in Table 2. Among respondents with acute diarrheal illness who sought medical advice, our outpatient clinic of pediatric specialized hospital and the private clinics were the most common first sought health care providers been found in 37% in both conditions. The main reasons for visiting our hospital is the efficient knowledgeable provider and that it is a well-known hospital. Seeking care was postponed for more than 3 days after onset of symptoms of diarrhea in 20.2% of cases.

**Table 2:** Health care seeking pattern among studied group

Characteristics		%
First sought provider	CUH*	37.4
	Private clinic	36.6
	Govern. Hospital	13.8
	NGO**	5.5
	Primary health care	2.9
	Pharmacy	1.3
	HIO (Health Insurance Organization)	0.5
	Others	1
CUH is the	2^nd^ provider	33.7
	3^rd^	13.6
	4^th^	6.8
	5^th^	3.5
Causes of visiting CUH	Efficient Knowledgeable provider	62.9
	Near service	4.2
	Well-known hospital	41.2
	Low cost	20.1
	Others	13.1
Perception about CUH services	v. good	33.1
	Fair	62.7
	Bad	4.2
Waiting time satisfaction	v. good	37.4
	Fair	47.7
	Bad	14.9
Provider communication satisfaction	v. good	50.6
	Fair	47.5
	Bad	1.9
Health education for prevention given		34.2

* CUH: Cairo university hospital

** NGO: Non-governmental organizations

The total tangible costs of seeking care are shown in Table 3. Regarding the intangible costs, the mean transport time consumed was 3.6±10.9 hours and the mean waiting time was 27.5±44 7 minutes.

**Table 3:** Total tangible costs of seeking healthcare among studied group

	Cost	Mean±SD	Median	Interquartile range
Direct costs(US$)	Provider	6.6±3.84	2.65	3.8(5-1.2)
	Medications	8.7±11.8	5.8	8.3(10.7-2.5)
	Investigations(Laboratory& Radiological)	1.3±6.5	0.2	0.3(0.5-0.16)
	Tips	0.2±0.5	0.3	0.3(0.16-.016)
	Hospital	1.1±6.2	0.16	0.16(0.3-0.16)
	Total direct costs	13.2±19.5	8.6	12.9(16.2-3.3)
Indirect costs(US$)	Transport	2.*7±*30.9	0.66	1.3(1.6-0.3)
	Cost due to absence from work	10.6±21.9	3.3	11.8(12-0.16)
	Total indirect costs	11.3±93.1	1.66	6.8(7.3-0.5)

The main cost items that parents suffer are medications cost and the cost due to absence from work being US$8.7±11.8 (35.5% of total tangible costs) and US$10.6±22 (43% of total tangible costs), respectively. The mean cost per diarrheal episode is US$24.5 which almost consumes 29.5% of the mean monthly income. The mean income was estimated as exactly below the poverty line US$83 per month).

Regarding the insurance status among study population, awareness about insurance was present in 73% of cases; however coverage was only in 43%. Among the insured children only 45.7% utilizes these services. Willingness to pay for insurance (WTP) among non-insured children was in 70% of cases. The mean affordable contribution/family member/month was US$1.6 among insured and US$1.44 among non-insured. All medical services would be desired by 53.4% of the interviewees.

## Discussion

Diarrheal disease is a major cause of death in children in the developing world. In developing countries a quarter of infant and childhood mortality is related to diarrhea [5]. Diarrhea creates huge burden on families of low income countries as Egypt. This study is a hospital based study aiming to knowing the direct and indirect costs of diarrhea so we can help to alleviate that burden of the disease on the Egyptian families.

About 95% of our patients were less than five years old and those less than one year represent 39. 6%. This shows a strong tendency of diarrhea to occur among children less than 2 years of age. This finding is consistent with other studies conducted in the Middle East countries and other various developing countries, where the major burden of diseases due to rotavirus occurs in the first and second year of life [6–8].

About 90% were of low and middle socioeconomic standard, with a median monthly family income of US$83. This was similar to a similar study from Brazil which showed that socioeconomic factors contributed to determining diarrhea occurrence as children who lived under more precarious socioeconomic conditions (in shack-type housing or in a family that did not own more than four items) are more likely to have diarrhea [9]. This might be due to unsafe water supply and reduced frequency of healthy habits like washing hands and objects.

The main reasons for visiting our public teaching hospital is the efficient knowledgeable provider and that it is a well-known hospital despite of low cost of user fees. This goes with a similar study from Benin and Guinea where the poor have a tendency to use the public health centers to a larger extent than the rich [10]. In agreement with previous studies, we found that the choice of one type of care over another may depend on characteristics of the services (e. g. perceived quality of care, accessibility [11].

These costs of diarrhea were exceptionally high and catastrophic in Egypt as the mean income was estimated to be US$83 per month, and the total tangible costs of the diarrheal disease will cost the family about US$24.5 which represents almost 29.5% of the family income in that month. This is similar to what observed in Cambodia where the Cambodia Demographic and Health Survey in 2005 indicated that the direct costs of treatment were high. The calculations, included transportation, food, medication, and administrative, pathology and other fees, indicating that the average cost of a single illness episode was US$15.52 for public facilities, US$18. 62 for private services, and US$6.25 for non-medical services the mean income was estimated as exactly on the poverty line (of US$13.50 per month). People often had to finance health spending through out-of-pocket payments and loans, or sold property, goods or labor to meet the costs. Costs were often catastrophic, exacerbating the extreme poverty of those least able to afford it [3]. In Georgia, average health care costs constituted 9.1% of total household income for a household. The largest share of total expenditures was hospital care (46%) followed by medicines (26%) [12]. However, in our study, largest share of total expenditures was are and the cost due to absence from work (43%) and medications cost (35. 5%).

Seeking care was postponed for more than 3 days after onset of symptoms of diarrhea in 20.2% of cases. This was found in spite of severity of illness as indicated by the finding that about three fourths of the cases were associated with fever and vomiting. This delay might affect the progress of the disease and affect the outcome. In their study, Terra de Souza et al found that poor or delayed care-seeking has been identified as a contributor in up to 70% of child deaths [11]. It is known that a higher monetary price for health care tends to reduce, or at least delay, health service use, especially among the poor, unless accompanied by improvements in service quality [13]. The cycle of poverty and poor health requires a balance of interventions from public health professionals, environmentalists, and people working in areas that greatly affect health (e. g., labor, trade, and agriculture). We must focus on the health consequences of poverty. By doing so, we can break the cycle of poverty leading to ill health and ill health leading to poverty [14].

Regarding the insurance status among study population, awareness about insurance was present in 73% of cases; however coverage was only in 43%. Regardless of location, poor people are more likely than affluent people to lack health insurance [15]. Reasons for skipping health insurance should be investigated. We found that having insurance does not always protect families from high out-of-pocket health care spending.

## Conclusion

Diarrhea causes great socio-economic burdens for families in Egypt, which could result in significant delay in seeking health care. It is essential for decision makers to consider the possibility of changing the systems of application for health insurance, given the families’ needs to improve the quality of health care at public health facilities.
